# Microfabrication of a micron-scale microbial-domestication pod for *in situ* cultivation of marine bacteria†

**DOI:** 10.1039/d2ra05420e

**Published:** 2022-10-03

**Authors:** Sydney K. Wheatley, Christopher Cartmell, Elias Madadian, Sara Badr, Bradley A. Haltli, Russell G. Kerr, Ali Ahmadi

**Affiliations:** Faculty of Sustainable Design Engineering, University of Prince Edward Island 550 University Avenue Charlottetown PE C1A 4P3 Canada ali.ahmadi@etsmtl.ca; Department of Mechanical Engineering, École de technologie supérieure (ÉTS) Montreal QC H3C 1K3 Canada; Department of Chemistry, University of Prince Edward Island 550 University Avenue Charlottetown PE C1A 4P3 Canada; Nautilus Biosciences Croda, Regis and Joan Duffy Research Centre 550 University Avenue Charlottetown PE C1A 4P3 Canada; Department of Biomedical Science, University of Prince Edward Island 550 University Avenue Charlottetown PE C1A 4P3 Canada

## Abstract

Through the hyphenation of microfabrication, microfluidics and microbiology, we report the development of a μMicrobial-Domestication Pod (μMD Pod). This *in situ* cultivation device facilitates cell signaling from neighbouring species and interactions with environmental stimuli for marine bacterial growth to overcome current barriers faced by standard laboratory cultivation methods.

## Abstract 2

Through the hyphenation of microfabrication, microfluidics and microbiology, we report the development of a μMicrobial-Domestication Pod (μMD Pod) for *in situ* cultivation of bacteria.

## Graphical abstract

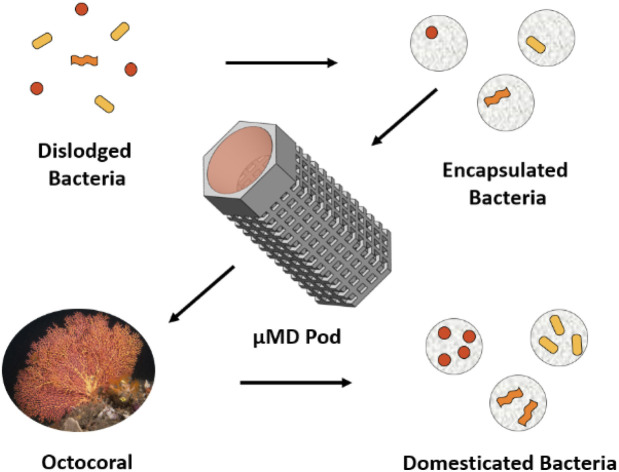

## Introduction

‘The Great Plate Count Anomaly^1^’ is one of the most prevalent mysteries within microbiology^2^ which illustrates the impracticality of cultivating the vast majority of microbial species under standard laboratory cultivation techniques.^3,4^ Despite the ambiguity of the root cause, a few theories suggest that the uncultivability of microbes stems from the deficiency of chemical signaling from neighboring microbes in single cell cultures, a loss of physiological stimuli such as pressure and temperature changes,^5^ or prolonged dormancy.^6,7^

For over 40 years, natural products have proven to be an unrivalled source for therapeutic agents.^8^ As antibiotic resistant strains of microbes increase, so too does the need for the discovery of new microbial species.^9^ The development of novel antibiotic therapeutics must not be overlooked as deaths resulting from antimicrobial resistance are currently around 700 000 people globally each year, with a projected increase to 10 million people by 2050 if no further action is taken.^10^ Despite the relevancy of microbes, we find ourselves in an era described as the discovery void^11,12^ with rediscovery and replication of known compounds presenting an increasing issue. Whilst developing methods such as molecular networking has shown promising results in defeating the quandary of replication,^13^ access to the missing chemical space is still unobtained.

One method developed to combat ‘The Great Plate Count Anomaly’ is the use of diffusion chambers. The success of the diffusion chambers has been reported to be 300 times greater for the formation of micro-colonies in comparison to traditional plating techniques.^14,15^ Diffusion chambers consist of a hollow structure, sealed with filter membranes, which are then situated within the natural environment for *in situ* incubation. This *in situ* cultivation allows for cell-to-cell communication and physiological stimuli changes; however, this process requires serial dilution to extinction in order to purify colonies, which significantly lowers the throughput characteristics of the device.

**Fig. 1:**
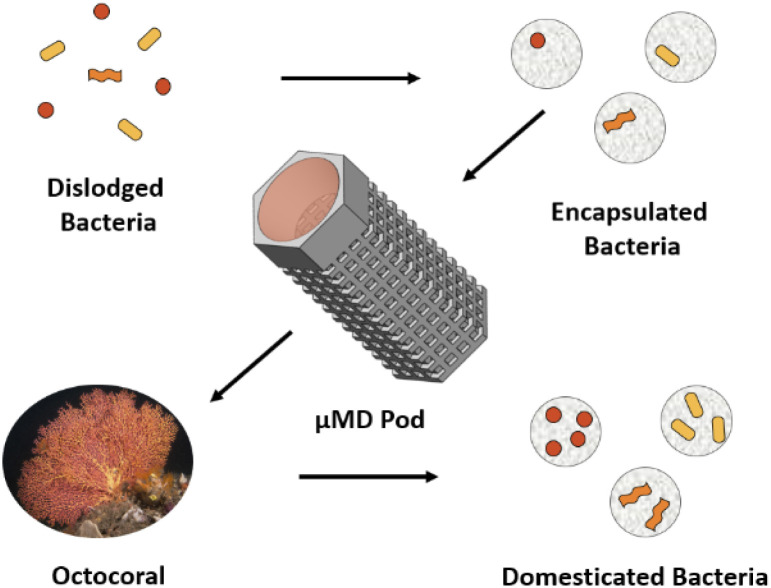
The process of *in situ* marine bacteria cultivation using the μMD Pod.

The isolation chip, more commonly referred to as the iChip,^16^ is a simplistic, low-tech device which yields great potential as proved through the isolation of *teixobactin* from the novel bacterium *Eleftheria terrae* in 2015.^17^ The iChip was an evolution of the diffusion chamber which intended to increase the throughput by individually sorting a single bacterium into its own individual well. Although vastly improving throughput, this method may be viewed as a single cell approach in which co-culture could be diminished through the lack of cell-to-cell communication within the individual wells. Our previous work introduced the concept of the Microbial Domestication Pod (MD Pod),^18^ a device allowing for single cell isolation while providing the opportunity for cell-to-cell communication. The preparation of the MD Pod was achieved using a microfluidic set up to encapsulate a single cell within an agarose microbead. The agarose microbeads were then inserted into the MD Pod which was used for *in situ* marine sediment cultivation. The MD Pod featured filter membranes which facilitated the entry of nutrients while preventing the entry of other microbes. However, although the original MD Pod was suitable for the *in situ* cultivation within marine sediment, its size prevented its use within many invertebrates. This study aimed to improve on our current methodology while producing a device capable of *in situ* cultivation within small marine invertebrates^19^ (Fig. 1). Marine invertebrates have long been considered an exciting source of marine natural products,^20,21^ often however, it is not the invertebrate itself which is responsible for the production of the therapeutic agent, but instead, a bacterial species living in symbiosis.^22^ Previous studies in the Kerr lab have demonstrated how *in situ* cultivation within marine invertebrates can be a fruitful source of new compounds with the use of a modified iChip. However, neither the iChip nor the original MD Pod is suitable for *in situ* cultivation in many smaller species of marine invertebrates and as such, development of a micro-scale device was necessary.

As mentioned, the microbial diversity living within marine invertebrates, octocorals in particular, shows great promise for the discovery of new natural products. However, no previous work has been capable of exploring the *in situ* cultivation of bacteria within such a confined area. A novel approach is needed to access these resources while facilitating the diffusion of nutrients and chemical signaling. Herein we report the production of the μMD Pod, a device microfabricated through two photon polymerization yielding high-resolution micron-sized^23^ features negating the use of fragile membranes. This work maintains the benefits of a “domestication” period in the bacterium's natural environment simultaneously harnessing the natural symbiotic relationships present through co-cultivation within the nursery of the microbeads,^24^ re-opening doors for bioprospecting marine invertebrate-associated natural products.

## Materials and methods

### μMD Pod fabrication

**Fig. 2:**
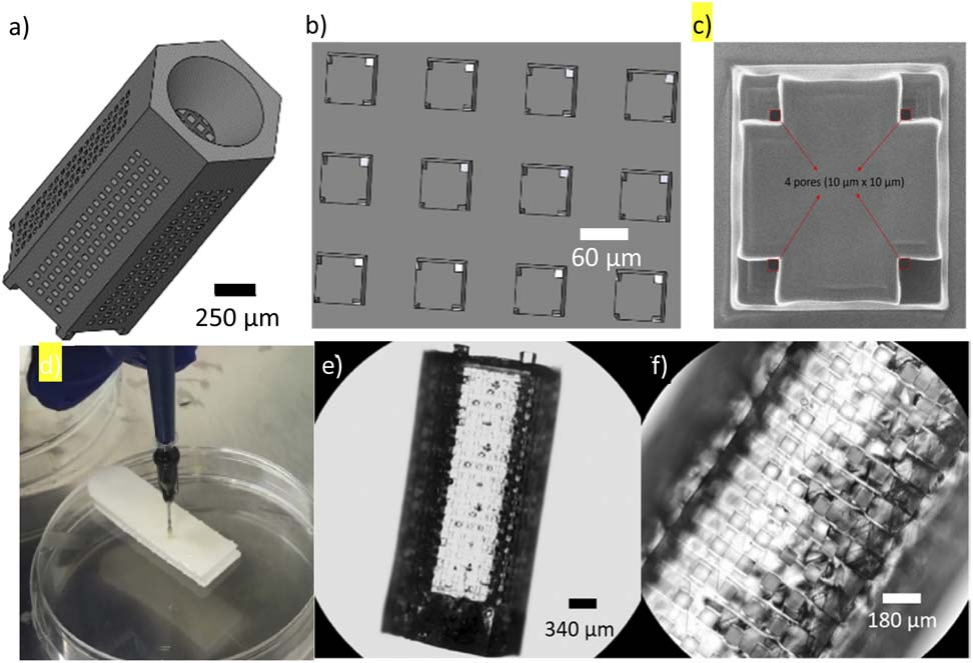
(a) μMD Pod CAD design; (b) μMD Pod CAD designs displaying the overlapping walls; (c) an HiM image of 5 μm pores in overlapping wall; (d) loading of the μMD Pod; (e) microscopic image of fabricated μMD Pod; and (f) a close-up of the pores in the μMD Pod.

The μMicrobial Domestication Pod (μMD Pod) was designed using Solidworks (Dassault Systems, 2020). The design featured two layers of grid-like walls with 60 μm square openings periodically separated in a 60 μm array. The two layers were overlapped with a slight offset to yield 10 μm square through-holes (Fig. 2a–c). The top of the Pod featured a 500 μm diameter tapered hole for inserting the microbead suspension into the cavity of the Pod. The internal cavity of the 2.850 mm μMD Pod was 1.74 μL which can contain over 1500 microbeads of 200 μm diameter. The base of the Pod had seven octagonal pedestals required for the post-fabrication removal process off the silicon slide. The design was printed using two-photon polymerization technology (Nanoscribe GmbH, Germany). The device was fabricated out of IP-S resin, a biocompatible, non-cytotoxic material used in microfluidic applications. The exposure time for a batch of five μMD Pods was 24 hours using the IP-S resin. Once printed, the Pods were autoclaved before being loaded with the microbead suspension.

To fabricate such a small device, different aspects of the previous devices were taken into consideration. The use of membranes was present in all previous *in situ* cultivation devices but presented many challenges due to its fragility leading to tears or improper sealing. In this study, two-photon polymerization (Nanoscribe GmbH, Germany) was used to negate the use of membranes and achieve the desired micron-sized features. The application of two-photon polymerization enabled the fabrication of a porous device that could facilitate chemical signaling and nutrient diffusion. The μMD Pod featured an open-top for the loading of the cell-encapsulated microbeads. The μMD Pods were then sealed using natural wax (Michaels, USA). Natural wax was selected for sealing of the device as it is a biocompatible material^25^ that is suitable for a saltwater environment and inherently quick drying.

### μMD Pod seal testing

Three μMD Pods were aseptically loaded using a 21-gauge sterilized needle with microencapsulated *Sphingomonas phyllosphaerae* which were produced using a previously reported microfluidic technique^18^ (Fig. 2d). This bacterium was selected due to its distinct yellow pigmentation. Each of these μMD Pods were sealed using a natural wax by using forceps to dip the opening of the pod into the wax heated to 70 °C and set aside until dry. Once sealed, the μMD Pods were then submerged in Marine Broth 2216 (Difco) diluted to 10% strength (dMB) and incubated at room temperature for 7 days without shaking. Three control cultures were performed simultaneously which consisted of *S. phyllosphaerae* suspended in 10% marine broth.

### Incubation of encapsulated *S. phyllosphaerae* microbeads

Production of microbeads encapsulating *S. phyllosphaerae* were produced as previously reported.^18^ An encapsulation of *Streptomyces* sp. was also performed using the same method. The microbeads were suspended within a dialysis bag. The incubation within the dialysis bag was performed to ensure that no outside bacterial communities could gain access to the microbeads whilst allowing nutrients to cross the membrane. The microbeads were incubated within the aquarium for a period of 7 days. After completion of the incubation period, the microbeads were removed and stained using DMAO in order to assess microbial growth within the microbeads.

### Staining for microbead bacterial growth

After the completion of the incubation period, the μMD Pod was sacrificed onto a glass slide and the debris was washed with 10 μL of sterile water to remove any surface bound microbeads. Cell viability assessments were performed using fluorescent Live/Dead bacterial staining (PromoCell, Germany). For the Live/Dead assay, a ratio of 100 : 1 bacterial sample to stain mixture (prepared according to the manufacturer's recommendation) was conducted and viability was assessed by fluorescence microscopy using a Revolve4 microscope and a 20× objective (ECHO, USA). The microbeads were imaged both prior and post incubation to examine colony growth.

## Results and discussion

### μMD Pod fabrication

After printing of the μMD Pods, the printability number was found to be 1.05 ± 0.03 (Pr = *L*^2^/16*A*) where *L* is the perimeter of the pore and *A* is the area of the pore^26,27^ (Fig. 2c, e and f). The pedestals on the base of the Pods provided the stability needed during fabrication and transport as the first shipment of Pods arrived damaged. The new Pods were successfully fabricated and shipped; however, the Pods have an exposure time of 24 hours per five Pods and the manipulation of these devices proved to be a challenge as the IP-S resin was quite brittle. Future studies will investigate the use of the IP-PDMS and IP-Visio resins which will yield a more flexible device. Additionally, the current design featured 10 μm by 10 μm pores; however, we have shown that smaller pore sizes are attainable for this device using two-photon polymerization (Fig. 2c). As concluded from the pressure simulation (details provided in ESI†), the recommended maximum pressure on the μMD Pods to avoid breakage is 350 kPa, which can allow the pods to reach depths of 35 m in saltwater.

### μMD Pod seal testing

During this experiment, dMB was used as the culture medium to allow for a clear indication of outgrowth if the Pods were not successfully sealed. *S. phyllosphaerae* was microencapsulated in agarose beads of 250 μm. The Pods were then incubated for 7 days at room temperature before comparison against control cultures. During this trial, bacterial growth could be observed in dMB for the control cultures due to the visible change in broth colour. However, no outgrowth was observed in cultures inoculated with the μMD Pods sealed with sealing wax as there was no visible change in the broth, nor was there any colony formation for the plated broth. Therefore, it was confirmed that the μMD Pod pores are successfully fabricated and the inlet is successfully sealed.

### μMD Pod insertion into coral and microbial *in situ* cultivation

**Fig. 3:**
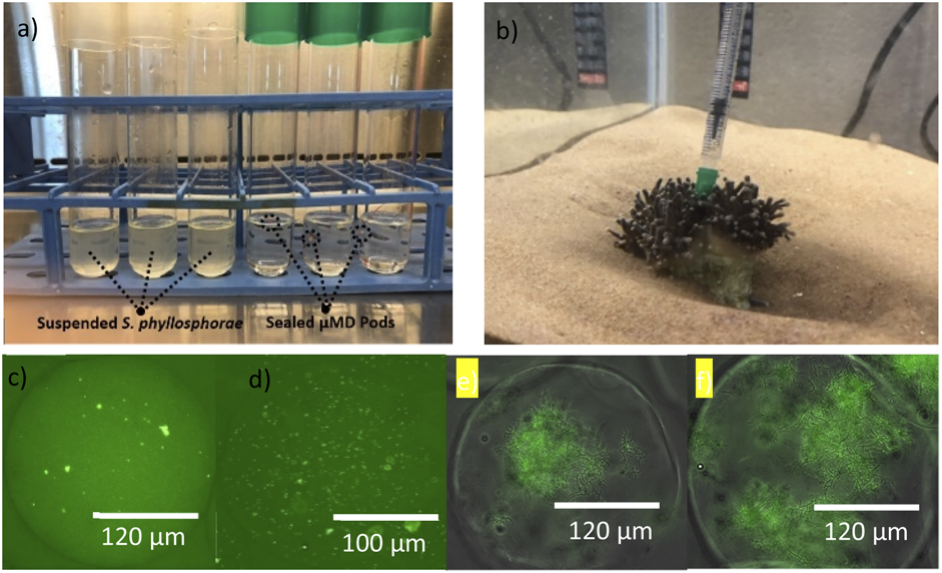
(a) Control cultures of *S. phyllosphaerae* in triplicate (left) and sealed μMD Pods in triplicate showing no outgrowth (right); (b) attempted insertion of μMD Pod into *Euphyllia glabrescens*; (c) microbead bacterial content before incubation within an aquarium; (d) microbead bacterial content post incubation within an aquarium; (e) microbead containing *Streptomyces* sp. before incubation; (f) microbead containing *Streptomyces* sp. post incubation.

To assess the application of this technique for *in situ* incubation applications within marine invertebrate hosts, a salt-water aquarium was set up containing the coral *Euphyllia glabrescens* (AFK Reef Supplies, Canada). In order to assess the mechanical properties of the μMD Pod it was shown that the μMD Pod can be inserted into a coral (Fig. 3b). However, insertion of the μMD Pod into corals with limited coenenchyma whilst possessing large polyps was observed to be challenging. The coenenchyma is the tissue of the coral that connects the colony of polyps.^26^ It is within this region that the transfer of nutrients between polyps occurs, and it is hypothesized to contain diverse microbial communities.^26,27^

*S. phyllosphaerae* containing microbeads were incubated for a period of seven days to assess bacterial growth within the beads in a marine environment (as described in the Methods). Following incubation, the microbeads were retrieved, and the agarose microbeads were stained using DMAO. A significant increase in the number of colonies growing within the microbead post incubation was observed, indicating that the bacteria multiplied during *in situ* incubation (Fig. 3c and d). This experiment was also performed using *Streptomyces* sp. (RKND-216), a marine bacterium, which also experienced a significant increase in bacterial growth following the incubatory period (Fig. 3e and f).

Upon previous successful trials for the isolation and cultivation of marine bacteria, the need to reduce the size of the Pods was identified to discover new bacteria in different environments. The μMD Pod is currently in its preliminary proof-of-concept stage; however, future iterations will have to overcome the current fabrication limitations relating to the co-dependency of the overall device size and smallest pore size. It has been observed that Octocorallia contain diverse communities of bacteria.^28,29^ However, this resource has not yet been fully explored due to the physical limitations present. The primary impediment which has prevented *in situ* cultivation within octocorals is attributable to the small branches of the corals. In the case of *Antillogorgia elizabethae*^30,31^ and *Antillogorgia bipinnata*,^32^ two species of interest, the branch thicknesses range from 1–2 mm. Given the limited space, an altered approach was developed in this proof-of-concept study demonstrating with technological advances two-photon microfabrication presents a viable approach to enable access to the untapped resource of novel natural products within such octocorals.

## Conclusions

We have successfully demonstrated how two-photon polymerization printing can be used as a method of fabrication of *in situ* cultivation devices. Whilst the technology is currently lacking the resolution to create pore sizes of <0.1 μm allowing for the removal of any membranes, this proof on concept displays great potential. The slow growing *S. phyllosphaerae* was successfully microencapsulated within agarose beads and through the use of live/dead imaging, bacterial growth was observed within the μMD Pod. As technology advances, we believe this is an approach which will provide the opportunity to gain access to currently obtainable microbial dark matter, harnessing synergistic relationships between marine invertebrate associated bacteria and environmental stimuli. This has the potential to be a powerful process for gaining access to new taxa of bacteria, in return, providing access into much needed therapeutic agents. Additionally, the μMD Pod's small size provides great potential creating the possibility of *in situ* cultivation of ‘unculturable’ bacteria within the human microbiome. However, future studies must be conducted to expand the capabilities of the device by reducing its overall size and pore size.

## Conflicts of interest

The authors declare no conflicts of interest.

## Supplementary Material

RA-012-D2RA05420E-s001
