# Supplementary material for: Microfabrication of a micron-scale microbial-domestication pod for in situ cultivation of marine bacteria
Source: RSC Adv. 2022 Oct 3;12(43):28123–7. doi: 10.1039/d2ra05420e (PMC9527566; doi:10.1039/d2ra05420e)
Supplement: RA-012-D2RA05420E-s001 [file RA-012-D2RA05420E-s001.pdf]

Electronic Supplementary Material (ESI) for RSC Advances.
This journal is © The Royal Society of Chemistry 2022

Supplementary information

Microfabrication of a Micron-scale Microbial-Domestication Pod for *In-Situ* Cultivation of Marine Bacteria

Sydney K. Wheatley[a,b†], Christopher Cartmell[c†], Elias Madadian[a,b], Sara Badr[a,b], Bradley A. Haltli[d,e], Russell G. Kerr[c,d,e] and Ali Ahmadi[a,b,e*]

[a] *Faculty of Sustainable Design Engineering, University of Prince Edward Island, 550 University Avenue, Charlottetown, PE, C1A 4P3, Canada.*
[b] *Department of Mechanical Engineering, École de technologie supérieure (ÉTS), Montreal, QC, H3C 1K3, Canada*
[c] *Department of Chemistry, University of Prince Edward Island, 550 University Avenue, Charlottetown, PE, C1A 4P3, Canada.*
[d] *Nautilus Biosciences Croda, Regis and Joan Duffy Research Centre, 550 University Avenue, Charlottetown, PE, C1A 4P3, Canada.*
[e] *Department of Biomedical Science, University of Prince Edward Island, 550 University Avenue, Charlottetown, PE, C1A 4P3, Canada.*
† Sydney K. Wheatley and Christopher Cartmell are considered joint first authors on this publication.

Bacterial Encapsulation

Escherichia coli K12 ER2925 (New England Biolabs) was cultured in 10 ml of Luria-Bertani (LB) Miller broth (EMD Millipore Sigma) at room temperature, shaking at 200 rpm for 18 h. Optical density at 600 nm (OD_{600}) was measured using a NanoDrop® ND-1000 Spectrophotometer (Thermo Fisher Scientific).
Sphingomonas phyllosphorae was cultured in 10 ml of marine broth (EMD Millipore Sigma) at room temperature, shaking at 200 rpm for 48 h before being subjected to microfluidic encapsulationwhich was performed as reported in Alkayyali *et al*[1]

Model µMD Seal Testing

To determine an appropriate sealing material and method of sealing, a large-scale model of the µMD pod was used. The large-scale pod was designed on Solidworks (Dassault Systems, 2020) and printed using a stereolithography 3D printer (Form 3, FormLabs, United States). This non-porous model was 9 mm tall and featured a 1.5 mm open cavity for top loading of the sample, and a shape similar to the µMD Pod. The internal volume of the large-scale pod was 25.8 µL.

Seal testing was performed in triplicate against standard cultivation techniques. For ease of handling, a larger scale model of the µMD pod was 3D printed with an internal volume of 5 µL. All tests were performed in triplicate, the 3D printed µMD Pods were sterilized via autoclaving and inoculated with 5 µL of *E. coli*. The pods were sealed in and added to fermentation tubes containing LB medium. Controls were performed by inoculating 5 µL of *E. coli* into unsealed 3D printed µMD Pods in fermentation tubes containing LB medium as well as an additional three tubes of LB medium inoculated with 5 µL of *E. coli*. During the sealing tests, if outgrowth was observed in the surrounding medium that the µMD Pod was submerged in, the sealing was deemed unsuccessful (FigS1. a). Two methods were trialled for the sealing of the µMD Pods. The first method involved the synthesis of a photocurable liquid polystyrene as previously reported[2]. For this experiment, 20 µL of the liquid polystyrene was pipetted over the opening of the 3D printed pod and subjected to photo-curation for 1 min using a halogen lamp. Although initially promising, over time the polystyrene seal absorbed liquid, and its integrity became compromised causing outgrowth within the liquid medium. The second method for sealing consisted of using wax. After loading of the pods with *E. coli*, the pod was dip sealed with the sealing wax. All tests were performed over 7 days with no outgrowth observed for the wax sealed 3D printed µMD Pods.

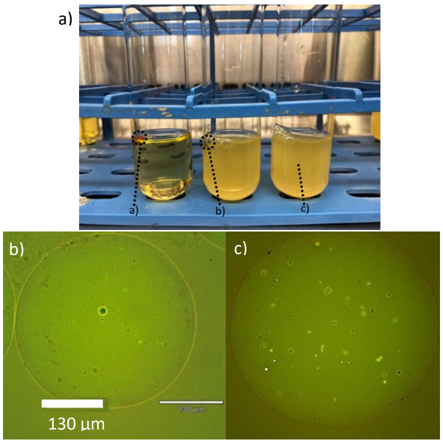

FigS1. a) Sealed model µMD Pod inoculated with *E. coli* showing no outgrowth; b) unsealed model µMD Pod inoculated with *E. coil*; c) control LB media inoculated with *E. coli*.

μMD Pressure Simulation

The designed model was imported in ABAQUS/EXPLICIT software, and the finite element model was created using 176363 C3D10 elements. A homogeneous section for material was assigned and the material properties for IP-S was provided by Nanoscribe as 20 MPa and 0.3 for Young's modulus and Poisson's ratio respectively. Additionally, for the yield stress, the estimated amount of σ_y ~ 20 MPa was used. As shown in Fig S2, a tie boundary condition was applied to one end of the model and a uniform pressure was applied on one of the faces of the pod to examine the maximum pressure that can be applied to the pod till the model reaches the yield stress.

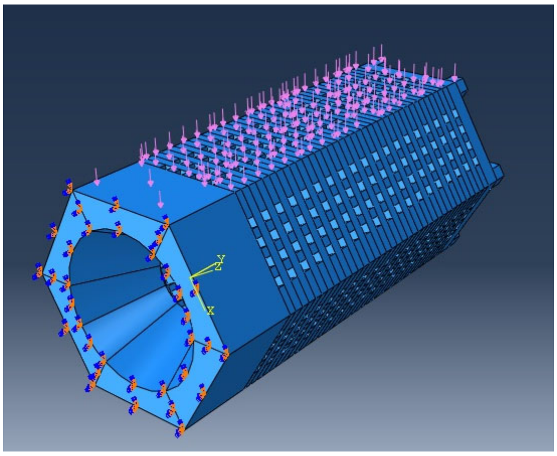

Fig S2 The finite element model of the μMD Pod indicating boundary conditions and applied pressure.

Fig. S3 a) shows the location of the maximum stress in the model and an enlarged image of the section that bears the maximum stress. As expected, the edges of the pore that border the solid base of the pod are under a higher stress considering the structure of the model, the stress concentration, and the lower thickness of the model at those locations. Fig. S3 a) also indicates a maximum principal stress of σ = 19.5 MPa resulting from the applied pressure of 350 kPa. As this stress is approaching the yield stress of the resin, we consider this as the maximum pressure that should be applied on the pods. Additionally, the maximum displacement of the model, with the given pressure, was measured to be 7.99 μm at the free end of the pod which can be observed in Fig. S3 b).

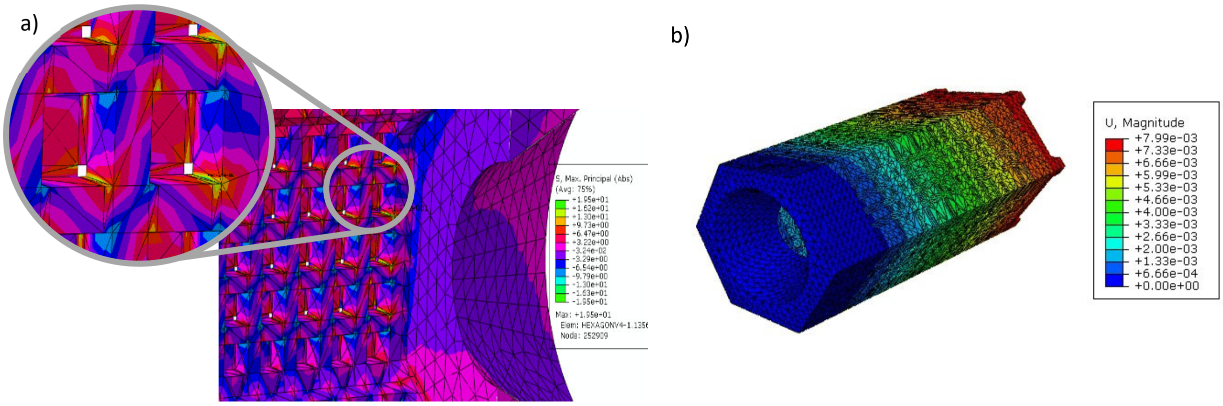

Fig. S3a) A visual model of the stressed regions within the μMD Pod; b) A visual model of the displacement of the μMD Pod under pressure.

1. Alkayyali, T.; Pope, E.; Wheatley, S. K.; Cartmell, C.; Haltli, B.; Kerr, R. G.; Ahmadi, A. *Biotechnol. Bioeng.* 2021, ***118***, 1166-1176.
2. Nargang, T. M.; Brockmann, L.; Nikolov, P. M.; *et al. Lab on a Chip*. 2014, ***14***, 2698-2708
